# Trends and birth outcomes in adolescent refugees and migrants on the Thailand-Myanmar border, 1986-2016: an observational study

**DOI:** 10.12688/wellcomeopenres.14613.1

**Published:** 2018-05-21

**Authors:** Amber L. Parker, Daniel M. Parker, Blooming Night Zan, Aung Myat Min, Mary Ellen Gilder, Maxime Ringringulu, Elsi Win, Jacher Wiladphaingern, Prakaykaew Charunwatthana, François Nosten, Sue J. Lee, Rose McGready

**Affiliations:** 1 Shoklo Malaria Research Unit, Mahidol-Oxford Tropical Medicine Research Un, Mahidol University, Mae Sot, 63110, Thailand; 2 Department of Population Health and Disease Prevention, Program in Public Health, University of California, Irvine, Irvine, California, USA; 3 Karen Refugee Committee, Mae Sot, Thailand; 4 Mahidol-Oxford Tropical Medicine Research Unit, Mahidol University, Bangkok, 10400, Thailand; 5 Department of Clinical Tropical Medicine, Faculty of Tropical Medicine, Mahidol University, Bangkok, 10400, Thailand; 6 Centre for Tropical Medicine and Global Health, Nuffield Department of Medicine Research Building, University of Oxford, Oxford, OX3 7FZ, UK

**Keywords:** adolescent pregnancy; teenage pregnancy; reproductive health; birth outcomes; pregnancy trend; pregnancy outcomes; refugee health; migrant health

## Abstract

**Background:** Currently there are more adolescents (10-19 years old) and young adults (20-24 years old) than ever. Reproductive health among this age group is often overlooked, although it can have a profound impact on the future. This is especially the case in conflict zones and refugee settings, where there is a heightened need for reproductive health care, and where both the resources and possibility for data collation are usually limited.

**Methods:** Here we report on pregnancies, birth outcomes and risk factors for repeat pregnancies among adolescent and young adult refugees and migrants from antenatal clinics on the Thailand-Myanmar border across a 30 year time span.

**Results:** Pregnancy and fertility rates were persistently high. Compared with 20-24-year-olds, 15-19-year-olds who reported being unable to read had 2.35 (CI: 1.97 – 2.81) times the odds for repeat pregnancy (gravidity >2). In primigravidae, the proportion of small for gestational age (SGA) and preterm births (PTB), and neonatal deaths (NND) decreased with increasing maternal age (all p <0.001). After adjustment, this association retained significance for PTB (cut-off point, ≤18 years) but not for SGA and NND.

**Conclusions:** There is considerable room for improvement in adolescent pregnancy rates in these border populations, and educational opportunities may play a key role in effective interventions.

## Introduction

The population of the current generation of young people (10–24 years of age) is the largest in the history of humanity; these individuals are at a critical phase in terms of achieving their potential and securing the future of the next generation ^1^. The health and wellbeing of this age group has important ramifications for the future of nations, regions and the world ^2^. However in low- and middle-income countries, as well as areas of conflict, limited resources and technical abilities have resulted in a widespread state of neglect with regard to adolescent reproductive health ^3^.

A recent in-depth report on the world’s adolescents showed that more than 90% of adolescent births occur in low- and middle-income countries ^4^. These pregnancies are found disproportionally in rural areas of the developing world, where girls are twice as likely to be married before 18 years of age than their urban counterparts, and those with no education carry a three-fold higher risk of pregnancy than those with a secondary or higher education ^4^. Pregnancy and childbirth in adolescence have been associated with negative health outcomes, such as low birth weight (LBW), small for gestational age (SGA), preterm birth (PTB), low Apgar scores, and neonatal and maternal mortality ^5– 8^. However, these negative health associations are not seen in all populations; in some cohorts they have lower risk for important obstetric complications, such as cesarean delivery ^9^.

Marriage and pregnancy in adolescence are influenced by a host of environmental factors, including local socio-cultural norms, the availability of contraceptives, education, socio-economic status and occupational opportunities. In high-income countries, where there are abundant educational and employment opportunities, the socio-economic impact of these pregnancies is substantial, as pregnancy and parenthood often limit educational and career trajectories of young people. In settings where livelihoods are based predominantly on unskilled labor and higher education is uncommon, the opportunity cost of early pregnancy may be lower.

In conflict zones and refugee settings, the situation is further complicated by limited resources for health and education, contraceptive availability, legal status and security, and by cultural and religious influences. Protective community support mechanisms, which make adolescent parenthood socially viable in some agricultural societies, are also often lost in migration or mass population movements ^10– 12^. Refugee camps on the Thailand-Myanmar border have now been in existence for decades and multiple generations of refugees have lived their entire lives in this setting.

As a result of difficulties in obtaining and maintaining accurate population census data in the often chaotic environments of refugee camps, data on adolescent pregnancy and birth outcomes are seldom reported ^13^, with most publications arising from countries of resettlement ^14, 15^. The objective of this research was to evaluate trends in adolescent (10–19 years of age) pregnancies, including birth outcomes and risk factors for repeat pregnancy among refugees and migrants along the Thailand-Myanmar border.

## Methods

### Refugee populations

Maela Camp is the largest remaining refugee camp on the Thailand-Myanmar border. Its current population is approximately 37,000 and it has existed since the early 1990s, when over 30 smaller camps were consolidated ^16^. Karen, ethnic Burman, and other minority ethnic groups from Myanmar make up the camp population.

Shoklo Malaria Research Unit (SMRU) began providing maternal health care in 1986 in Shoklo Refugee Camp, and later moved with the population to Maela Camp. SMRU established antenatal clinics (ANCs) because of a very high malaria-related maternal mortality ratio (estimated at >1,000 per 100,000 live births) in the camp ^17, 18^, and continued to provide antenatal care and delivery services for three decades.

### Migrant populations

The Thai economy is more developed and stable than most of its neighbors in the Greater Mekong Subregion, and migrants from neighboring nations (especially Myanmar) gravitate to the possibility of higher-paying jobs ^19, 20^. In Tak Province, Thailand, a large proportion of migrants are unregistered, live in temporary shelters that move with the planting or harvesting seasons and have poor access to health services. In collaboration with the Thai Ministry of Public Health ^21^, SMRU established fever clinics, which have since treated tens of thousands of migrants for malaria ^22^, including many pregnant women at ANCs established in late 1998.

### Data and definitions

Data on pregnancies came from SMRU ANC files, which recorded all pregnant women attending ANCs. The ANC data include self-reported gravidity and ability to read (a proxy for education), age, residency status (refugee or migrant), smoking and pregnancy outcomes. Ability to read was consistently collected beginning in 2011. Body mass index was reported for women who attended in first trimester (defined as <14 weeks’ gestation). Control of malaria in this setting has relied on active screening by blood-smear microscopy at ANC visits, with screening frequency determined by malaria prevalence and all episodes treated according to WHO guidelines. Anaemia was defined as at least one haematocrit <30% diagnosed through active screening at ANC. Eclampsia was defined as convulsions with hypertension (≥140/90) and pre-eclampsia as hypertension with proteinuria confirmed by at least two assessments 6 hours apart. Maternal deaths included women who died while pregnant and up to 6 weeks post-partum. Post-partum haemorrhage was defined as estimated blood loss of 500 ml or more after delivery.

Women were welcome to register and participate in antenatal care even if they planned delivery elsewhere. When this occurred, it was recorded as an unknown pregnancy outcome. Staff encouraged women to deliver at a staffed and equipped facility although the tradition (or necessity) in the early years was homebirth ^23^. Progress of labor was monitored using the WHO partogram, and women requiring cesarean section were referred to the nearest Thai government hospital where this service was available. Staff weighed infants as soon as possible after clinic or home birth, and those presenting within 72 hours of life were included in birth weight analysis. Newborns were weighed using electronic scales (Seca medical scales were used, with a precision of 5 g), and LBW was classified as birth weight <2500g. Miscarriages were defined as delivery before 28 weeks gestation and SGA was defined using international standards, with a birth weight below the 10th percentile for estimated gestational age and sex ^24^. PTBs were those occurring before 37 weeks’ gestation, the majority determined by ultrasound assessment at the first ANC provided free of charge by locally trained sonographers ^25^. NNDs were deaths that occurred within 28 days of birth, in the context of cohort studies with dedicated infant follow-up ^26^.

For the refugee camp, population estimates were available from The Border Consortium (TBC) monthly food registries, beginning in 1998. Estimates from December of each year were used for this analysis. In 1996, Médecins Sans Frontières conducted a full population census of Maela Camp. The age and sex structure from this census was used in combination with total population estimates (from TBC) to estimate the total female adolescent (10–14 and 15–19 years old) and young adult (20–24 year old) population for each year. The population size and age structure of migrant populations contributing to these data are unknown, and therefore fertility rates were not estimated for migrants.

### Analysis

The proportion of all pregnancies among refugees and migrants attributed to females 15–19 years of age were calculated from the ANC records from 1986 through 2016. The migrant clinics opened in late 1998, therefore this analysis uses migrant data from 1999 onwards. Proportions were calculated as the total number of pregnancies for each age group divided by the total number of pregnancies for each residency status. For the refugee population only, age-specific fertility rates (ASFR) were calculated using live births with an estimated gestational age of at least 28 weeks in refugee females in combination with population estimates for each respective age group based on TBC reports ^27^.

Pregnancy- and childbirth-related outcomes were compared between the 15-19- and the 20-24-year-old groups using the Student’s t-test or Mann-Whitney U-test for continuous data and the chi-squared or Fisher’s exact test for categorical data. Pregnancies in the 10-14-year-old group were not included in outcome analysis due to low numbers (n=70).

Risk factors for PTB, SGA and NND were analyzed for primigravida women, with age as the main covariate. Using 24-year-olds as the reference group, the risk for each year of age was quantified using logistic regression and adjusted for BMI (BMI<18.5 kg/m ^2^), malaria during pregnancy, year of birth, residency status, and ANC attendance in the first trimester. NND was additionally adjusted for preterm births. Risk factors for repeat pregnancy (gravidity ≥2) among adolescents (15–19-year-olds) were analyzed using logistic regression (n=2 repeat pregnancies among 10–14-year-olds excluded). Covariates included self-reported ability to read, years of age, residency status, and year (ordinal, in three year groups, e.g., 1986–1989, 1990–1992, etc.) Separate regressions were therefore run for the effect of: A) time and age on repeat pregnancy among adolescent refugees (1986 through 2016); B) time, age and residency status on repeat pregnancy among adolescent refugees and migrants (1999 through 2016); and C) time, age, residency status and ability to read, on repeat pregnancy among adolescent refugees and migrants (2011 through 2016).

All statistical analyses were done using STATA v14.1 (STATA Corp) and R v3.4.0 ^28^.

### Ethics statement

For the extraction of data, ethical approval for retrospective analysis of pregnancy records was given by the Oxford Tropical Research Ethics Committee (OXTREC 28–09, amended 19 April 2012) and by the local Community Advisory Board TCAB 4/1/2015.

## Results

**Figure 1.**
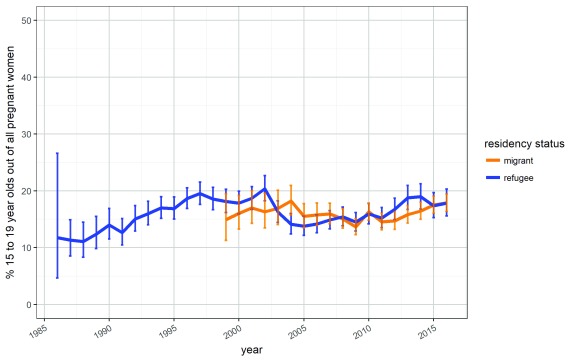
Proportion of all pregnancies attributed to 15–19-year-olds by year and residency status. Proportion was calculated as the total number of pregnancies among 15–19-year-olds divided by the total number of pregnancies for all ages. Wilson binomial confidence intervals are included.

Between 1986 and 2016, SMRU ANCs registered 72,662 pregnancies. Of these, 70 (0.096%) were in the 10–14-year-old group (6 were 13 years and 64 were 14 years), 11,838 (16.3%) were in the 15–19-year-old group, and 20,475 (28.2%) were in the 20–24 year old age group. The proportion of registered adolescent pregnancies remained relatively stable across the 30-year time frame for refugees, and over the 17-year time frame for migrants ( Figure 1).

### Age-specific fertility

**Figure 2.**
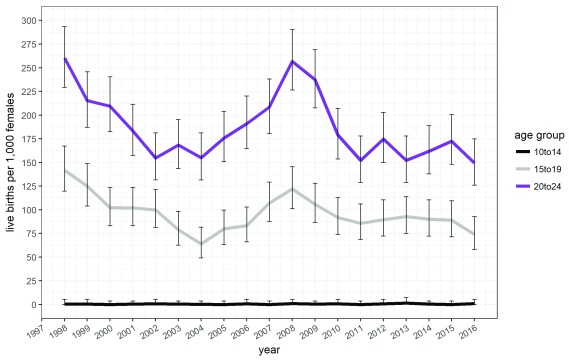
Age-specific fertility rates and Poisson confidence intervals in refugees (1998–2016).

Among refugees, for whom population data were available, age-specific fertility fluctuated over time, especially during the time period from 2005 to 2008 when there was an increase in fertility rates for both the 15–19- and the 20–24-year-old age groups ( Figure 2). Fertility in the 15–19-year-old age group was at its highest in 1998 at approximately 142 (95% CI: 120-167), peaked again in 2008 at 122 (95% CI: 101-146) and then decreased to 74 (95% CI: 58-93) per 1000 women by 2016. Similar trends were observed for women in the 20–24-year-old age group.

### Pregnancy characteristics at enrollment and morbidity

Characteristics and pregnancy morbidity of attendees in the 15–19- and 20–24-year-old group were summarized and expected differences were confirmed for gravidity and BMI ( Table 1). Unexpected differences included a significantly higher first trimester ANC attendance among 15–19-year-olds (48.5%) than 20–24-year-olds (45.8%, p<0.001); and a lower proportion of smoking in pregnancy among 15–19-year-olds ( Table 1). The 15–19-year-old group had a higher proportion of malaria detected in pregnancy (18.8% vs. 15.3%, p<0.001) but the proportion of any anaemia, eclampsia or pre-eclampsia, and post-partum haemorrhage were not significantly different (all p>0.10, Table 1).

**Table 1.** Pregnancy outcomes for all ANC attendees, by age group (15–19 or 20–24 years of age) in 1986–2016. Data are n (%) unless otherwise stated.

	Sample size	15–19 years of age	20–24 years of age	p-value
Total	n=32,313	n=11,838	n=20,475	
*Baseline*				
Nulliparous (G1P0)	32,219	8870/11,800 (75.2)	7294/20,419 (35.7)	<0.001
Can read	12,399	2919/4526 (64.5)	5070/7873 (64.4)	0.914
Attend ANC in 1st trimester	31,494	5595/11,532 (48.5)	9141/19,962 (45.8)	<0.001
Underweight (BMI<18.5 kg/m ^2^)	20,572	1124/7455 (15.1)	1761/13,117 (13.4)	0.001
Smoking	26,202	916/9627 (9.5)	2602/16,575 (15.7)	<0.001
*Pregnancy morbidity*				
Malaria in pregnancy	32,303	2226/11,831 (18.8)	3138/20,472 (15.3)	<0.001
Anaemia in pregnancy	30,231	3692/11,091 (33.3)	6501/19,140 (34.0)	0.230
Eclampsia/pre-eclampsia	30,666	137/11,193 (1.2)	212/19,473 (1.1)	0.282
Maternal deaths	32,221	16/11,805 (0.1)	16/20,416 (0.1)	0.117
Post-partum haemorrhage	19,710	273/7293 (3.7)	513/12,417 (4.1)	0.179
*Pregnancy outcome ^1, 2^*				
Miscarriage	25,962	649/9507 (6.8)	1156/16,455 (7.0)	0.544
Gestational age ≥28 weeks	25,962	8811/9507 (92.7)	15256/16,455 (92.7)	0.918
Twins ^3^	24,067	45/8811 (0.5)	106/15,256 (0.69)	0.081
Singleton ^3^	24,067	8751/8811 (99.3)	15121/15,256 (99.1)	0.089
*Place of birth ^3, 4^*				
Home	21,128	1709/7793 (21.9)	3360/13,335 (25.2)	<0.001
SMRU clinic	21,128	5372/7793 (68.9)	8743/13,335 (65.6)	<0.001
Hospital	21,128	630/7793 (8.1)	1076/13,335 (8.1)	0.969
*Instrumental delivery*				
Not recorded ^5^	23,872	533/8751 (6.1)	1125/15,121 (7.4)	<0.001
Cesarean section	22,214	244/8218 (3.0)	452/13,996 (3.2)	0.282
Vacuum delivery	22,214	129/8218 (1.6)	187/13,996 (1.3)	0.156
Forceps delivery	22214	18/8218 (0.2)	26/13,996 (0.2)	0.590
*Singleton outcomes ^6^*				
Median (IQR) gestation, weeks	23,872	39.1 (37.6, 39.6)	39.2 (38.2, 40.1)	<0.001
PTB, all	23,872	1342/8751 (15.3)	1465/15,121 (9.7)	<0.001
PTB, EGA by ultrasound	13,557	776/5038 (15.4)	698/8519 (8.2)	<0.001
Proportion stillbirth	23,818	85/8733 (1.0)	135/15,085 (0.9)	0.542
Major congenital abnormality	23,569	118/8650 (1.4)	214/14,919 (1.4)	0.659
Valid birth weight ^7^	22,946	7476/8422 (88.8)	12,713/14,524 (87.5)	0.005
Mean (SD) birth weight, g	20,189	2810 (465)	2938 (453)	<0.001
Low birth weight	20,189	1402/7476 (18.6)	1618/12,713 (12.7)	<0.001
SGA	20,013	2101/7419 (28.3)	2941/12,594 (23.4)	<0.001
Neonatal death ^8^	12,224	118/4472 (2.6)	114/7752 (1.5)	<0.001

^1^Reliable measure of gestation missing n=819 (n=306 among 15–19-year-olds and n=513 among 20–24-year-olds). ^2^Registered to antenatal care but birth outcome unknown n=5532 (2025 among 15–19-year-olds and 3507 among 20–24-year-olds). ^3^Includes infants with estimated gestational age of ≥28 weeks. ^4^Delivered but birth place unrecorded, n=2744. ^5^Delivered but presentation e.g cephalic or breech, missing n=1658. ^6^Includes singletons live/still born, normal or congenitally abnormal, gestational age ≥28 weeks. ^7^Excludes stillbirth, congenital abnormality and birth weight not measured in the first 72 h of life. ^8^Only reliably recorded from study cohort data. ANC, antenatal clinic; PTB, preterm birth; LBW, low birth weight; SGA, small for gestational age.

### Birth outcomes

The proportion of missing data for gestational age and unknown outcome of pregnancy (usually due to movement out of the area) were similar between age groups, as was the proportion of miscarriage and twin pregnancy (p > 0.05 for all, Table 1). Compared to 20–24-year-olds, a lower proportion of the 15–19-year-old age group birthed at home (21.9% vs. 25.2%, p <0.001), resulting in a higher proportion delivering with skilled birth attendants in the SMRU clinic (68.9% vs. 65.6%, p <0.001). There was no significant difference in the proportion of women from each age group who delivered in the hospital or who required cesarean section or instrumental (vacuum or forceps) delivery (both p >0.10, Table 1).

There was a significantly higher proportion of PTB (15.3% vs. 9.69%, p <0.001), LBW (18.6% vs. 12.7%, p <0.001), SGA (28.3% vs. 23.4%, p<0.001) and NND (within cohort studies, 2.64% vs. 1.47%, p<0.001) in adolescents compared to young adults. There was no difference in stillbirth or major congenital abnormality. The proportion of SGA, PTB and NND were compared for each year of age from 15 through 24 years in primigravidae. SGA was analyzed in lieu of LBW as it provides a better summation of poor birth weight. The proportion of SGA, PTB and NND decreased as age increased, and was a significant trend (p=0.008, p<0.001 and p<0.001, respectively). The decrease in proportion of SGA by year of age was not significant after adjustment for BMI (BMI <18.5 mg/kg ^2^), malaria during pregnancy, year of birth, residency status, and first trimester ANC attendance ( Supplementary Table 1). NND followed a similar pattern ( Supplementary Table 2). The decreasing trend in PTB with increasing age remained significant for 15-, 16-, 17- and 18–year-olds when compared with 24–year-olds (cut-off point ≤18 years). Residency status and birth in later years were associated with reduced adjusted odds of PTB ( Table 2). Although the maternal mortality ratio was higher in the 15–19-year-old age group, the difference was not significant ( Table 1).

**Table 2.** Proportion of preterm births in primigravida women aged 15–24 years.

	N	Full term, n (%)	Preterm, n (%)	Univariate p-value *	AOR (95%CI), p-value
Age					
15	316	244 (77.2)	72 (22.8)	<0.001, 9 df	2.730 (1.722-4.327), p<0.001
16	853	701 (82.2)	152 (17.8)		1.850 (1.239-2.765), p=0.003
17	1,459	1,222 (83.8)	237 (16.2)		1.524 (1.045-2.224), p=0.029
18	2,139	1,800 (84.2)	339 (15.9)		1.663 (1.160-2.384), p=0.006
19	1,801	1,568 (87.1)	233 (12.9)		1.191 (0.820-1.729), p=0.359
20	1,934	1,693 (87.5)	241 (12.5)		1.014 (0.695-1.480), p=0.944
21	977	860 (88.0)	117 (12.0)		1.111 (0.741-1.666), p=0.612
22	960	842 (87.7)	118 (12.3)		1.032 (0.682-1.563), p=0.880
23	873	782 (89.6)	91 (10.4)		0.850 (0.549-1.316), p=0.466
24	579	513 (88.6)	66 (11.4)		Reference
Underweight (BMI 18.5 kg/m ^2^) **					
Yes	986	851 (86.3)	135 (13.7)	0.188	1.182 (0.965-1.449), p=0.106
No	6682	5866 (87.8)	816 (12.2)		Reference
Malaria ^†^					
Yes	2100	1732 (82.5)	368 (17.5)	<0.001	1.091 (0.880-1.352), P=0.426
No	9786	8489 (86.8)	1297 (13.3)		Reference
Attended ANC in trimester 1					
Yes	5039	4835 (87.0)	654 (13.0)	0.005	0.900 (0.779-1.040), p=0.152
No	6852	5840 (85.2)	1012 (14.8)		Reference
Residency status					
Refugee	7790	6584 (84.5)	1206 (15.5)	<0.001	0.783 (0.676-0.908), p=0.001
Migrant	4101	3641 (88.8)	460 (11.2)		Reference
Year of birth ^††^					
1986–2016	6496	6259 (97.9)	137 (2.11)	<0.001, 30 df	
2004–2016	4691	4627 (98.6)	64 (1.36)	<.0001	0.925 (0.887-0.965), p<0.001

*Chi-squared p-value; **BMI available from 2004 only; ^†^at any time during pregnancy; ^††^from 1986 to 2016 for univariate, from 2004 for adjusted analysis. AOR, adjusted odds ratio; BMI, body mass index; ANC, antenatal clinic; df, degrees of freedom.

### Risk factors for repeat pregnancies

In total, 2 of the 70 females in the 10–14-year-old age group reported having repeat pregnancies, both were 14–year-olds (one refugee, one migrant). Repeat pregnancies were reported among 25% (2,930/11,800) of 15–19-year-olds. This proportion was 26% (1693/6429) among refugees, and 22% (955/4365) among migrants (p<0.0001).

The self-reported ability to read was higher among pregnant refugees than migrants (74% vs. 59%, p<0.0001). Among refugee women in the 10-14-year-old age group, 92% (11/12) reported ability to read, higher than in 15–19-year-olds (76%; 1,021/1,344) and 20–24-year-olds (76%; 1,620/2,120), (p = 0.5009). Among migrant women, 50% (7/14) of 10–14-year-olds, 64% (63/1,349) of 15–19-year-olds and 62% (2,484/4,015) of 20–24-year-olds, reported the ability to read (p = 0.2339).

There was no obvious long-term trend (between 1986 and 2016) in repeat pregnancies among refugee adolescents ( Table 3, model A). Repeat pregnancies peaked between 1996 and 2004, but otherwise remained relatively stable across the 30-year time period. There was an apparent decrease in repeat pregnancies among refugees and migrants in 2008 through 2016, when compared to the 1999-2001 time period ( Table 3, model B). Inability to read was strongly associated with repeat pregnancy ( Table 3, model C). Females in the 15–19-year-old age group who reported being unable to read had twice the odds (adjusted OR, 2.35; CI, 1.97–2.81) of having repeat pregnancies. As expected, the odds of having repeat pregnancies sharply increased with age regardless of residency status and ability to read ( Table 3, models A–C).

**Table 3.** Risk factors for repeat pregnancy (gravidity ≥2) among adolescents (15–19 years old). Not all variables are available for all time periods. Residency status is only available since 1999, when migrant clinics were opened and literacy was recorded beginning in 2011. Model A includes only refugees, time and age (both as ordinal variables); Model B also includes residency status (categorical); Model C also includes self-reported literacy (yes/no).

	Model A (n = 6459)			Model B (n = 8733)			Model C (n = 3558)		
	AOR	95% CI	p-value	AOR	95% CI	p-value	AOR	95% CI	p-value
Date									
1986–1989	Comparison								
1990–1992	1.69	(1.08-2.69)	0.0229						
1993–1995	1.41	(0.93-2.18)	0.1102						
1996–1998	1.93	(1.29-2.94)	0.0019						
1999–2001	1.73	(1.15-2.65)	0.0107	Comparison					
2002–2004	1.56	(1.03-2.41)	0.0396	0.96	(0.71-1.04)	0.7081			
2005–2007	1.37	(0.91-2.12)	0.1404	0.86	(0.65-0.93)	0.1157			
2008–2010	1.31	(0.87-2.02)	0.2039	0.78	(0.64-0.89)	0.0063			
2011–2013	1.25	(0.77-2.05)	0.3770	0.61	(0.47-0.79)	0.0001	Comparison		
2014–2016	1.06	(0.71-1.62)	0.7868	0.56	(0.47-0.67)	<0.0001	0.92	(0.73-1.16)	0.4890
Age, years									
15	Comparison			Comparison			Comparison		
16	1.66	(0.96-3.06)	0.0858	1.63	(1.03-2.71)	0.0465	2.15	(0.92-5.88)	0.1000
17	2.84	(1.71-5.10)	0.0002	2.68	(1.75-4.33)	<0.0001	3.61	(1.66-9.45)	0.0033
18	5.78	(3.52-10.26)	<0.0001	4.73	(3.12-7.56)	<0.0001	6.19	(2.93-16.00)	<0.0001
19	10.31	(6.28-18.30)	<0.0001	8.25	(5.45-13.18)	<0.0001	11.28	(5.35-29.06)	<0.0001
Migrant				Comparison			Comparison		
Refugee				1.16	(1.05-1.29)	0.0051	1.62	(1.36-1.94)	<0.0001
Can read							Comparison		
Cannot read							2.35	(1.97-2.81)	<0.0001

* Chi –squared p-value; ** BMI available from 2004 only; †at any time during pregnancy; ††1986–2016 for univariate, from 2004 for adjusted analysis. AOR, adjusted odds ratio.

## Discussion

These data indicate relatively high pregnancy and fertility rates among young refugees and high but fluctuating ASFR in 15–19-year-old refugees over three decades. Pregnancies and repeat pregnancies among migrants in this age range were also common, suggesting that this isn’t just a refugee-specific scenario. Fertility in the 15–19-year-old age group (averaging 94 live births per 1,000 between 1998 and 2016) is comparable to levels in high-fertility regions in the developing world (91 in Ethiopia, 2008 and 109 in Malawi, 2009 ^29^). At least one report from Thailand indicated that fertility was highest among 19–year-olds, with an ASFR of 58.3 per 1,000; lower than that seen in this 15–19-year-old age group ^30^. Much lower fertility rates are reported in 15–19-year-olds from high income countries such as USA and the Netherlands (with 32.3 and 3.9 births per 1,000 women in 2015, respectively ^31^). Fertility in 2016 appears to have decreased from baseline rates in 1998, which may suggest a recent positive change in contraceptive uptake ( Figure 2).

SGA and NND were significantly higher in younger mothers, but the effect disappeared when important, likely mediating, factors were included in the analysis (such as mother’s BMI, health status ( Supplementary Table 1, Supplementary Table 2)) ^32^. PTB remained a significant adverse birth outcome for 15–18-year-olds although absolute numbers are relatively small. Unfortunately PTB is rarely preventable, with the only available intervention being education about symptoms and when to seek medical attention. PTB has long-term implications, given that the newborn survives infancy with an increased risk of non-communicable disease in adulthood, including both cardiovascular disease and type 2 diabetes ^33, 34^. This increased risk of ill-health and the resulting economic disadvantage may lead to an intergenerational impact of adolescent pregnancies and therefore is an important motivator to facilitate delayed age at first pregnancy. Prevention of adolescent pregnancy by the provision of highly effective family planning, uninhibited by user financial constraints or stigma, would require engagement of adolescents and other key community stakeholders ^35, 36^. The proxy for education in this analysis—the ability to read—reduced repeat pregnancy by about half and supports several other studies showing relationships between education and number of offspring ^37– 39^.

Pregnancies among the youngest age group (10–14-year-olds) were low and two-thirds of births among 15–19-year-olds were attributable to adolescents aged 18 and 19 years old. While there are clear benefits to postponing childbirth and marriage, these benefits also have to be weighed against socio-cultural norms and the contexts in which people live. Some negative outcomes related to pregnancy and childbirth may be mediated in settings where marriage and childbirth at younger ages (e.g. 18–19-years old) are socially acceptable and where social networks (i.e. extended family households) normally help young mothers care for their children ^40^. In this “natural fertility” setting, pregnancy and marriage in adolescence remain socially acceptable and services did not appear to discriminate against young mothers, reflecting the ‘normality’ of adolescent pregnancy.

Cultural barriers also complicate interventions for adolescents, as contraception use before a woman's first child is often discouraged ^41^ and there is confusion about the safety of effective methods for adolescents, even among healthcare providers. In this generally conservative culture, support from community leaders for reproductive health education amongst school-aged children has been lacking. These attitudes have been changing with new leadership, opening up opportunities to make an impact on this generation. Given that closure of the camps is thought to be inevitable and imminent ^42, 43^, there is a small window of opportunity to engage and empower youths for success and self-determination in the next decade before they face significant new, unpredictable challenges ^44^. This is in agreement with the United Nation’s Sustainable Development Goal (SDG3) to ensure universal access to sexual and reproductive health-care services for all, at all ages by 2030.

### Limitations

There are several limitations to this work. Most of the indicators and outcomes reported were prospectively measured, but the gravidity of two or more and ability to read were self-reported. However, the outcome of each pregnancy is obtained during the obstetric history of each woman, reducing the risk for incorrect reporting of gravidity, and literacy rates remain in agreement with a previous survey on literacy in the population ^45^.

Estimations of numbers of women of reproductive age in the camp by different official organizations (namely the United Nations High Commission for Refugees (UNHCR) and TBC) do not concur ^13^. The analysis was based on the more inclusive TBC numbers, which are thought to best reflect the actual number of individuals residing in the camp. In 2008, when fertility rates in our analysis peak in all age groups, there is a high level of agreement between the estimates by the UNHCR and TBC. This peak also coincides with the largest resettlement in the history of the camps, where 16,607 persons were settled in third countries ^46^. The total numbers of people in the camp stayed relatively stable during this exodus as migration into the camp replaced those being resettled. There are no data on contraceptives and fertility rates prior to moving into the camp but access to antenatal services would have been limited and previous contraceptives supplies are likely to have been interrupted, potentially leading to the increase in ASFR. Once this population became settled in the camp, fertility rates appear to have decreased to normal camp levels.

## Conclusion

The refugee population described here has a high rate of pregnancies among adolescents, which has not changed significantly over the past three decades. Pregnancy among 10–14-year-olds is comparatively rare and the ability to read in the 15–19 year old age group appears to have a protective effect. Efforts at increasing educational opportunities may have widespread benefits for this population. The increased risk of PTB in 15–18 years of age can have impacts far beyond birth; influencing families, communities and even nations. There may be a short window of opportunity to provide interventions before this high-risk population is displaced once again, with renewed pressure for these refugee populations to move back to Myanmar, and an unprecedented openness among community leaders to facilitate change.

## Data availability

Due to ethical and security considerations, the data that supports the findings in this study can be accessed only through the Data Access Committee at Mahidol Oxford Tropical Medicine Research Unit (MORU). The data sharing policy can be found here: http://www.tropmedres.ac/data-sharing. The application form for datasets under the custodianship of MORU Tropical Network can be found in Supplementary File 1.

## Supplementary materials

**Supplementary Table 1. Proportion of small for gestational age in primigravida women aged 15–24 years.**

**Supplementary Table 2. Proportion of neonatal deaths in primigravida women aged 15–24 years.**

**Supplementary File 1. Data access form from Mahidol Oxford Tropical Medicine Research Unit.** The data sharing policy can be found here: http://www.tropmedres.ac/data-sharing.

## References

[1] Patton GC Sawyer SM Santelli JS: Our future: a *Lancet* commission on adolescent health and wellbeing. *Lancet.* 2016;387(10036):2423–2478. 10.1016/S0140-6736(16)00579-1 27174304 PMC5832967

[2] Blakemore SJ Mills KL: Is adolescence a sensitive period for sociocultural processing? *Annu Rev Psychol.* 2014;65:187–207. 10.1146/annurev-psych-010213-115202 24016274

[3] The World Bank: World Bank Report Development and the Next Generation, 2007 [Internet].2007 Reference Source

[4] Loaiza E Liang M: Adolescent pregnancy: A review of the evidence.New York;2013 Reference Source

[5] Ganchimeg T Ota E Morisaki N: Pregnancy and childbirth outcomes among adolescent mothers: a World Health Organization multicountry study. *BJOG.* 2014;121 Suppl 1:40–48. 10.1111/1471-0528.12630 24641534

[6] Chen XK Wen SW Fleming N: Teenage pregnancy and adverse birth outcomes: a large population based retrospective cohort study. *Int J Epidemiol.* 2007;36(2):368–73. 10.1093/ije/dyl284 17213208

[7] Scholl TO Hediger ML Salmon RW: Association between low gynaecological age and preterm birth. *Paediatr Perinat Epidemiol.* 1989;3(4):357–66. 10.1111/j.1365-3016.1989.tb00524.x 2587406

[8] Haiek L Lederman SA: The relationship between maternal weight for height and term birth weight in teens and adult women. *J Adolesc Health Care.* 1989;10(1):16–22. 10.1016/0197-0070(89)90041-7 2921184

[9] Kawakita T Wilson K Grantz KL: Adverse Maternal and Neonatal Outcomes in Adolescent Pregnancy. *J Pediatr Adolesc Gynecol.* 2016;29(2):130–136. 10.1016/j.jpag.2015.08.006 26327561 PMC4886236

[10] Mullany LC Lee TJ Yone L: Impact of community-based maternal health workers on coverage of essential maternal health interventions among internally displaced communities in eastern Burma: the MOM project. *PLoS Med.* 2010;7(8):1–11, e1000317. 10.1371/journal.pmed.1000317 20689805 PMC2914639

[11] Mcginn T Austin J Anfinson K: Family planning in conflict: results of cross-sectional baseline surveys in three African countries. *Confl Health.* 2011;5:11. 10.1186/1752-1505-5-11 21752241 PMC3162885

[12] IAWG: Inter-Agency Field Manual on Reproductive Health in Humanitarian Settings: 2010 Revision for Field Review.2010. 26203479

[13] Srikanok S Parker DM Parker AL: Empirical lessons regarding contraception in a protracted refugee setting: A descriptive study from Maela camp on the Thai-Myanmar border 1996 - 2015. *PLoS One.* 2017;12(2):e0172007. 10.1371/journal.pone.0172007 28231251 PMC5322876

[14] Gibson-Helm M Boyle J Cheng IH: Maternal health and pregnancy outcomes among women of refugee background from Asian countries. *Int J Gynecol Obstet.* 2015;129(2):146–151. 10.1016/j.ijgo.2014.10.036 25640714

[15] Gibson-Helm ME Teede HJ Cheng IH: Maternal health and pregnancy outcomes comparing migrant women born in humanitarian and nonhumanitarian source countries: a retrospective, observational study. *Birth.* 2015;42(2):116–124. 10.1111/birt.12159 25864573

[16] TBC: Between Worlds: Twenty Years on the Border [Internet].2004 Reference Source

[17] McGready R Boel M Rijken MJ: Effect of early detection and treatment on malaria related maternal mortality on the north-western border of thailand 1986-2010. *PLoS One.* 2012;7(7):e40244. 10.1371/journal.pone.0040244 22815732 PMC3399834

[18] Nosten F ter Kuile F Maelankirri L: Malaria during pregnancy in an area of unstable endemicity. *Trans R Soc Trop Med Hyg.* 1991;85(4):424–429. 10.1016/0035-9203(91)90205-D 1836685

[19] Acuin CS Khor GL Liabsuetrakul T: Maternal, neonatal, and child health in southeast Asia: towards greater regional collaboration. *Lancet.* 2011;377(9764):516–525. 10.1016/S0140-6736(10)62049-1 21269675 PMC7159081

[20] Acuin J Firestone R Htay TT: Southeast Asia: an emerging focus for global health. *Lancet.*Elsevier Ltd,2011;377(9765):534–535. 10.1016/S0140-6736(10)61426-2 21269684

[21] Carrara VI Sirilak S Thonglairuam J: Deployment of early diagnosis and mefloquine-artesunate treatment of falciparum malaria in Thailand: the Tak Malaria Initiative. *PLoS Med.* 2006;3(6):e183. 10.1371/journal.pmed.0030183 16719547 PMC1470664

[22] Carrara VI Lwin KM Phyo AP: Malaria Burden and Artemisinin Resistance in the Mobile and Migrant Population on the Thai-Myanmar Border, 1999-2011: An Observational Study. *PLoS Med.* 2013;10(3):e1001398. 10.1371/journal.pmed.1001398 23472056 PMC3589269

[23] White AL Min TH Gross MM: Accelerated Training of Skilled Birth Attendants in a Marginalized Population on the Thai-Myanmar Border: A Multiple Methods Program Evaluation. *PLoS One.* 2016;11(10):e0164363. 10.1371/journal.pone.0164363 27711144 PMC5053505

[24] Villar J Cheikh Ismail L Victora CG: International standards for newborn weight, length, and head circumference by gestational age and sex: The Newborn Cross-Sectional Study of the INTERGROWTH-21 ^st^ Project. *Lancet.* 2014;384(9946):857–868. 10.1016/s0140-6736(14)60932-6 25209487

[25] Rijken MJ Lee SJ Boel ME: Obstetric ultrasound scanning by local health workers in a refugee camp on the Thai-Burmese border. *Ultrasound Obstet Gynecol.* 2009;34(4):395–403. 10.1002/uog.7350 19790099 PMC3438883

[26] Carrara VI Stuetz W Lee SJ: Longer exposure to a new refugee food ration is associated with reduced prevalence of small for gestational age: Results from 2 cross-sectional surveys on the Thailand-Myanmar border. *Am J Clin Nutr.* 2017;105(6):1382–1390. 10.3945/ajcn.116.148262 28490508 PMC5445675

[27] The Border Consortium: The Border Consortium Annual Report 2016.2016 Reference Source

[28] R Core Team: R: A language and environment for statistical computing. R Foundation for Statistical Computing, Vienna, Austria.2018 Reference Source

[29] Sedgh G Finer LB Bankole A: Adolescent pregnancy, birth, and abortion rates across countries: levels and recent trends. *J Adolesc Health.* 2015;56(2):223–230. 10.1016/j.jadohealth.2014.09.007 25620306 PMC4852976

[30] Butchon R Liabsuetrakul T McNeil E: Birth rates and pregnancy complications in adolescent pregnant women giving birth in the hospitals of Thailand. *J Med Assoc Thai.* 2014;97(8):785–790. 25345252

[31] United States Census Bureau: International Data Base [Internet].2018; [cited 1 Jan 2017]. Reference Source

[32] Gibbs CM Wendt A Peters S: The impact of early age at first childbirth on maternal and infant health. *Paediatr Perinat Epidemiol.* 2012;26 Suppl 1:259–284. 10.1111/j.1365-3016.2012.01290.x 22742615 PMC4562289

[33] Barker DJ Hales CN Fall CH: Type 2 (non-insulin-dependent) diabetes mellitus, hypertension and hyperlipidaemia (syndrome X): relation to reduced fetal growth. *Diabetologia.* 1993;36(1):62–7. 10.1007/BF00399095 8436255

[34] Mericq V Martinez-Aguayo A Uauy R: Long-term metabolic risk among children born premature or small for gestational age. *Nat Rev Endocrinol.*Nature Publishing Group,2017;13(1):50–62. 10.1038/nrendo.2016.127 27539244

[35] Sarkar A Chandra-Mouli V Jain K: Community based reproductive health interventions for young married couples in resource-constrained settings: a systematic review. *BMC Public Health.* 2015;15:1037. 10.1186/s12889-015-2352-7 26452750 PMC4599316

[36] Gordon LP: Optimizing Adolescent LARC: an Answer to Pregnancy Prevention. *Ann Glob Health.* 2017;83(5–6):777–780. 10.1016/j.aogh.2017.11.001 29248094

[37] Adhikari R: Demographic, socio-economic, and cultural factors affecting fertility differentials in Nepal. *BMC Pregnancy Childbirth.* 2010;10:19. 10.1186/1471-2393-10-19 20426863 PMC2885993

[38] Seymour JW Frasso R Shofer FS: Cohort study of early literacy and childbearing over the reproductive lifecourse. *BMJ Open.* 2016;6(12):e013522. 10.1136/bmjopen-2016-013522 28039293 PMC5223742

[39] Thin Zaw PP Liabsuetrakul T McNeil E: Gender differences in exposure to SRH information and risky sexual debut among poor Myanmar youths. *BMC Public Health.* 2013;13:1122. 10.1186/1471-2458-13-1122 24304552 PMC4235033

[40] Kramer KL Lancaster JB: Teen motherhood in cross-cultural perspective. *Ann Hum Biol.* 2010;37(5):613–628. 10.3109/03014460903563434 20205610

[41] Salisbury P Hall L Kulkus S: Family planning knowledge, attitudes and practices in refugee and migrant pregnant and post-partum women on the Thailand-Myanmar border - a mixed methods study. *Reprod Health.* 2016;13(1):94. 10.1186/s12978-016-0212-2 27543078 PMC4992227

[42] Naing SY: Karen Refugee Committee Criticizes Refugee Repatriation Process. *The Irrawaddy.* 2016 Reference Source

[43] Downing J: In Thailand, waiting game continues for Myanmar refugees. *Frontier Myanmar.* 2016 Reference Source

[44] Areemit R Thinkhamrop J Kosuwon P: Adolescent pregnancy: Thailand's national agenda. *J Med Assoc Thai.* 2012;95 Suppl 7:S134–42. 23130445

[45] Carrara VI Hogan C De Pree C: Improved pregnancy outcome in refugees and migrants despite low literacy on the Thai-Burmese border: results of three cross-sectional surveys. *BMC Pregnancy Childbirth.* 2011;11:45. 10.1186/1471-2393-11-45 21679475 PMC3142536

[46] UNHCR: UNHCR Global Resettlement Statistical Report 2008 [Internet]. Washington, DC,2009 Reference Source

