# Supplementary material for: Trends and birth outcomes in adolescent refugees and migrants on the Thailand-Myanmar border, 1986-2016: an observational study
Source: Wellcome Open Res. 2018 May 21;3:62. [Version 1] doi: 10.12688/wellcomeopenres.14613.1 (PMC6039938; doi:10.12688/wellcomeopenres.14613.1)
Supplement: Supplementary file 1 [file wellcomeopenres-3-15910-s0000.tgz › 62952a39-c5d5-4c13-854c-79e485ef5fec.docx]

**Supplementary Table 1:** Proportion of small for gestational age in primigravida women aged 15-24 years

|  |  | **N** | **Normal weight for gestational age** | **Small for gestational age** | **Univariate p-value*** | **Adjusted Odds Ratio AOR (95%CI), p** |
| --- | --- | --- | --- | --- | --- | --- |
| **Age** |  |  |  |  |  |  |
|  | 15 | 263 | 181 (68.8) | 82 (31.2) | 0.022, 9 df | 1.095 (0.725-1.657), p=0.664 |
|  | 16 | 735 | 481 (65.4) | 254 (34.6) |  | 1.046 (0.759-1.443), p=0.782 |
|  | 17 | 1,241 | 874 (70.4) | 367 (29.6) |  | 0.911 (0.679-1.222), p=0.534 |
|  | 18 | 1,796 | 1,275 (71.0) | 521 (29.0) |  | 0.930 (0.705-1.226), p=0.605 |
|  | 19 | 1,572 | 1,137 (72.3) | 435 (27.7) |  | 0.965 (0.731-1.275), p=0.802 |
|  | 20 | 1,632 | 1,127 (69.1) | 505 (30.9) |  | 1.113 (0.858-1.490), p=0.381 |
|  | 21 | 841 | 612 (72.8) | 229 (27.2) |  | 0.996 (0.737-1.346), p=0.979 |
|  | 22 | 829 | 599 (72.3) | 230 (27.7) |  | 0.979 (0.721-1.329), p=0.892 |
|  | 23 | 770 | 551 (71.6) | 219 (28.4) |  | 1.107 (0.815-1.504), p=0.516 |
|  | 24 | 505 | 371 (73.5) | 134 (26.5) |  | Reference |
|  |  |  |  |  |  |  |
| Underweight (BMI 18.5 kg/m^2^)** | Yes | 849 | 571 (67.3) | 278 (32.7) | <0.001 | 1.584 (1.348-1.861), p<0.001 |
|  | No | 5831 | 4435(76.1) | 1396 (23.9) |  | Reference |
| Malaria† | Yes | 1596 | 1011 (63.4) | 585 (36.7) | <0.001 | 1.365 (1.138-1.638), P=0.001 |
|  | No | 8583 | 6193 (72.2) | 2390 (27.9) |  | Reference |
| Attend ANC tri 1 | Yes | 4407 | 3191 (72.4) | 1216 (27.6) | 0.002 | 0.886 (0.787-0.997), p=0.044 |
|  | No | 5777 | 4017 (69.5) | 1760 (30.5) |  | Reference |
| Residency status | Refugee | 7002 | 4895 (69.9) | 2107 (30.1) | 0.004 | 1.214 (1.076-1.370), p=0.002 |
|  | Migrant | 3184 | 2313 (72.7) | 869 (27.3) |  | Reference |
| Year of birth†† | 1986-2016 | 10,184 | 7208 (70.8) | 2976 (29.2) | <0.001, 30 df |  |
|  | 2004-2016 | 6667 | 4994 (74.9) | 1673 (25.1) | 0.006 | 0.945 (0.911-0.979), p=0.002 |

** Chi –squared p-value; ** BMI available from 2004 only; † at any time during pregnancy; †† from 1986 to 2016 for univariate, from 2004 for adjusted analysis*
