# Supplementary material for: Trends and birth outcomes in adolescent refugees and migrants on the Thailand-Myanmar border, 1986-2016: an observational study
Source: Wellcome Open Res. 2018 May 21;3:62. [Version 1] doi: 10.12688/wellcomeopenres.14613.1 (PMC6039938; doi:10.12688/wellcomeopenres.14613.1)
Supplement: Supplementary file 2 [file wellcomeopenres-3-15910-s0001.tgz › 8b637b75-eea1-4826-bb82-c4311d565026.docx]

**Supplementary Table 2:** Proportion of neonatal deaths in primigravida women aged 15-24 years

|  |  | **N** | **no neonate death** | **neonate death** | **Univariate p-value*** | **Adjusted Odds Ratio AOR (95%CI), p** |
| --- | --- | --- | --- | --- | --- | --- |
| Age |  |  |  |  |  |  |
|  | 15 | 147 | 140 (95.2) | 7 (4.8) | 0.003, 9 df | 6.983 (0.766-63.680), p=0.085 |
|  | 16 | 443 | 429 (96.8) | 14 (3.2) |  | 2.242 (0.240-20.937), p=0.479 |
|  | 17 | 737 | 713 (96.7) | 24 (3.3) |  | 6.106 (0.776-48.065), p=0.086 |
|  | 18 | 1,068 | 1,039 (97.3) | 29 (2.7) |  | 3.678 (0.470-28.755), p=0.215 |
|  | 19 | 1,029 | 1,010 (98.2) | 19 (1.9) |  | 2.449 (0.293-20.442), p=0.408 |
|  | 20 | 1,079 | 1,058 (98.1) | 21 (2.0) |  | 3.065 (0.377-24.904), p=0.295 |
|  | 21 | 580 | 572 (98.6) | 8 (1.4) |  | 1.370 (0.138-13.572), p=0.788 |
|  | 22 | 545 | 542 (99.5) | 3 (0.6) |  | 0.579 (0.035-9.477), p=0.702 |
|  | 23 | 519 | 511 (98.5) | 8 (1.5) |  | 3.227 (0.366-28.429), p=0.291 |
|  | 24 | 349 | 345 (98.9) | 4 (1.2) |  | Reference |
|  |  |  |  |  |  |  |
| Underweight (BMI 18.5 kg/m^2^)** | Yes | 620 | 610 (98.4) | 10 (1.61) | 0.602 | 1.279 (0.616-2.657), p=0.510 |
|  | No | 4073 | 4018 (98.7) | 55 (1.4) |  | Reference |
| Malaria† | Yes | 978 | 934 (95.5) | 44 (4.5) | <0.001 | 1.152 (0.553-2.402), P=0.706 |
|  | No | 5517 | 5425 (98.3) | 92 (1.7) |  | Reference |
| Attend ANC tri 1 | Yes | 2496 | 2449 (98.1) | 47 (1.9) | 0.317 | 0.703 (0.408-1.211), p=0.204 |
|  | No | 4000 | 3910 (97.8) | 90 (2.3) |  | Reference |
| Residency status | Refugee | 4113 | 4010 (97.5) | 103 (2.5) | 0.004 | 1.200 (0.701-2.056), p=0.506 |
|  | Migrant | 2383 | 2349 (98.6) | 34 (1.4) |  | Reference |
| Year of birth†† | 1986-2016 | 11891 | 102(86.0) | 1666 (14.0) | <0.001, 30df |  |
|  | 2004-2016 | 7655 | 6707 (87.6) | 948 (12.4) | <0.001 | 0.607 (0.489-0.753), p<0.001 |

** Chi –squared p-value; ** BMI available from 2004 only; † at any time during pregnancy; †† from 1986 to 2016 for univariate, from 2004 for adjusted analysis*
