# Supplementary material for: Trends and birth outcomes in adolescent refugees and migrants on the Thailand-Myanmar border, 1986-2016: an observational study
Source: Wellcome Open Res. 2018 May 21;3:62. [Version 1] doi: 10.12688/wellcomeopenres.14613.1 (PMC6039938; doi:10.12688/wellcomeopenres.14613.1)
Supplement: Peer review history

# Peer review: Referee response for version 1

**DOI:** 10.21956/wellcomeopenres.15910.r33369

**Published:** 2018-06-29

**Authors:** Emily Harville

**Affiliations:** 1 Department of Epidemiology, Tulane University, New Orleans, LA, USA

The manuscript presents occurrence and risk factors for adolescent pregnancy in a refugee and migrant populations near the Thai-Myanmar border. This is a valuable descriptive contribution as little epidemiology is available on this population.

 It would be helpful to know more about the differences between the migrant and refugee populations, including the likely size of the reproductive-aged female population. Do women return to their hometowns to give birth?

 Data are from the ANC files. How complete is attendance at ANC? Is the quality of the medical data likely to be equivalent for all groups? Will malaria, pre-eclampsia, and so on be equally well diagnosed and recorded? Will births be missed due to missing care?

 The first analysis presents adolescent pregnancy as a proportion of overall pregnancies. 30 years is a long time. How did the total number of pregnancies change? How were the general trends in the region?

 The second analysis presents age-specific fertility, for the refugee group, the one for which there is population denominator data. The age group sizes are estimated with TBC data – how accurate is this likely to be? Is there an explanation for the changes in age-specific fertility? Are the changes in age-specific fertility the same overall, or is an equivalent total fertility being shifted among different ages?  

 It would be useful to include a sensitivity analysis indicating how large an effect missing data or estimated data had on the results.

 Why was the analysis of neonatal death adjusted for preterm birth? PTB is likely a causal intermediate, not a confounder.

 Is there any information about pregnancies that do not end in birth?

 Minor point: the authors say age was taken from ANC files: is this age at pregnancy/ANC visit or age at birth?

I have read this submission. I believe that I have an appropriate level of expertise to confirm that it is of an acceptable scientific standard.

# Peer review: Referee response for version 1

**DOI:** 10.21956/wellcomeopenres.15910.r33201

**Published:** 2018-06-15

**Authors:** Jocelyn DeJong

**Affiliations:** 1 Epidemiology and Population Health Department, Faculty of Health Sciences, American University of Beirut (AUB), Beirut, Lebanon

This is an excellent article on an under-researched topic of birth outcomes of adolescent refugees and migrants on the Thailand-Myanmar border. Internationally there has been relatively little research on the reproductive health of refugees/migrants, particularly on birth outcomes. The study has benefited by the use of 30 years of data available through antenatal clinics offered by Shoklo Malaria Research Unit in collaboration with the Thai Ministry of Public Health. As noted by the authors, internationally it is difficult to collect data in refugee camps, and data on adolescent pregnancy and birth outcomes are not often reported.  Moreover, typically the smaller sample sizes of this age group present a challenge. This study uses clinic data over a thirty year period from 1986 to 2016, with variable availability of data as questions were added at a later date; the total number of pregnancies was 72,662 with about 16% of these being in the 15-19 age group of most interest.

 The study is careful and rigourous, and commendably cautious and transparent in the presentation of results. The article is very well written and easy to follow. 

 The authors, while reporting high levels of adolescent fertility, make a welcome caution that in a context of generalized high adolescent fertility, its negative social consequences and opportunity costs may be less than elsewhere. They note that there was no evidence among health-care workers of negative or discriminatory attitudes towards adolescents.

 The focus of the analysis overall is on adolescents and differences in age (with comparisons to the 20 to 24 age group), rather than refugee status per se. However, it would be useful to know more about this particular refugee and migrant study population. The article does not provide much information about the catchment population for the services. The fact that these services are provided by a malaria unit suggests that there may be a selection bias, with those with malaria more likely to use this service for ANC (malaria prevalence 18.8% in 15-19 age group and 15.3% in 20 -24 age group). Is this the main ANC service in this area or are there other services providing ANC and what is overall ANC use (if available)? Also information is needed for a reader not familiar with the context on the distinctions between refugees and migrants and the types of care provided to each group other than through this particular ANC service. Evidence presented suggests suggest that the migrants, being unregistered, are more disadvantaged (in terms of access to education, housing and access to health services) than the refugees, although repeat pregnancies are significantly higher among refugees. It seems that given that the refugee camps have existed for 30 years, many of this population would have been born in the camps and presumably are integrated in some ways into local communities. Therefore their status as a ‘refugee’ needs to be distinguished from the literature on short-term or medium-term refugees elsewhere.

 More comparison to the existing international literature on refugee/migrants would be welcome; only pregnancy and fertility rates and repeat pregnancy are compared to the literature. If no comparable studies are available, this should be noted. Are no data available to compare the results to the Thai local population?

 In the future, data on contraceptive use would be useful to collect as this is a limitation in the available data.

 Surprising findings – such as higher antenatal care utilization in first trimester among 15-19 year olds as compared to 20-24 year olds - need to be addressed in the discussion.

 Minor points – avoid contractions e.g. “isn’t” p. 7

I have read this submission. I believe that I have an appropriate level of expertise to confirm that it is of an acceptable scientific standard.
